# Non-Melanoma Skin Cancers: Biological and Clinical Features

**DOI:** 10.3390/ijms21155394

**Published:** 2020-07-29

**Authors:** Mauro Cives, Francesco Mannavola, Lucia Lospalluti, Maria Chiara Sergi, Gerardo Cazzato, Elisabetta Filoni, Federica Cavallo, Giuseppe Giudice, Luigia Stefania Stucci, Camillo Porta, Marco Tucci

**Affiliations:** 1 Section of Medical Oncology, Department of Biomedical Sciences and Clinical Oncology (DIMO), University of Bari ‘Aldo Moro’, 70121 Bari, Italy; mauro.cives@uniba.it (M.C.); francesco.mannavola@gmail.com (F.M.); sergimariachiara@gmail.com (M.C.S.); eli.filoni@gmail.com (E.F.); federica.cavallo91@gmail.com (F.C.); stuccistefania@gmail.com (L.S.S.); camillo.porta@uniba.it (C.P.); 2 National Cancer Center, Tumori Institute Giovanni Paolo II, 70121 Bari, Italy; 3 Section of Dermatology, Azienda Ospedaliero-Universitaria Policlinico di Bari, 70121 Bari, Italy; l.lospalluti@gmail.com; 4 Section of Pathology, University of Bari ‘Aldo Moro’, 70121 Bari, Italy; gerycazzato@hotmail.it; 5 Section of Plastic and Reconstructive Surgery, Department of Emergency and Organ Transplantation (DETO), University of Bari ‘Aldo Moro’, 70121 Bari, Italy; giuseppe.giudice@uniba.it

**Keywords:** skin cancer, basal cell carcinoma, squamous cell carcinoma, Merkel cell carcinoma, Hedgehog pathway, immunotherapy, anti-PD1 monoclonal antibodies

## Abstract

Non-melanoma skin cancers (NMSCs) include basal cell carcinoma (BCC), squamous cell carcinoma (SCC) and Merkel cell carcinoma (MCC). These neoplasms are highly diverse in their clinical presentation, as well as in their biological evolution. While the deregulation of the Hedgehog pathway is commonly observed in BCC, SCC and MCC are characterized by a strikingly elevated mutational and neoantigen burden. As result of our improved understanding of the biology of non-melanoma skin cancers, innovative treatment options including inhibitors of the Hedgehog pathway and immunotherapeutic agents have been recently investigated against these malignancies, leading to their approval by regulatory authorities. Herein, we review the most relevant biological and clinical features of NMSC, focusing on innovative treatment approaches.

## 1. Introduction

The incidence of skin cancers is increasing worldwide, as a result of the chronic exposure to sunlight, climatic changes and individual and social conditions [1,2]. As a whole, skin cancers include cutaneous melanoma (CM) and non-melanoma skin cancer (NMSC) that are mainly represented by basal cell carcinoma (BCC) and squamous cell carcinoma (SCC). A peculiar chapter in the context of skin cancers is represented by Merkel cell carcinoma (MCC), which is historically classified among neuroendocrine tumors, although its behavior resembles, in most instances, CM for the high propensity to colonize lymph nodes [3,4].

NMSC originates from epidermal cells and shows common epidemiology (e.g., higher prevalence in Caucasian subjects). On the other hand, MCC is thought to arise from Merkel cells and its frequency increases in equatorial geographic areas, particularly among subjects of white ethnicity. The pathogenesis of BCC, SCC and MCC is multifactorial, but skin exposure to physical carcinogens is the prevalent risk factor. Indeed, ultraviolet radiation (UVR) has the potential to directly drive the malignant transformation of progenitor cells [5,6,7,8]. Other risk factors [9,10,11,12] for the development of BCC and SCC include concurrent diseases and dedicated treatments (i.e., psoriasis), chronic exposure to human papilloma virus, drug-induced immune suppression in transplanted patients and targeted agents for the treatment of other cancer types (notably, melanoma). Several studies have also demonstrated that low social-economic status positively influences NMSC development [13,14]. The integration of the Merkel cell polyomavirus (MCP-yV) within the genome of tumor cells is a common event in MCC, and the molecular features leading to the MCPyV-induced malignant transformation of Merkel cells have recently been elucidated [15]. The deregulation of the Wnt/Hedgehog pathway has been described as a pivotal mechanism in the development of BCC while SCC and MCC are characterized by a high neoantigen burden [11,15,16,17,18,19,20,21]. The knowledge of the basic events driving NMSC has provided the basis for the development of new therapeutic strategies that include both targeted agents and immunotherapy.

Herein, we review the most relevant findings in NMSC and MCC focusing on clinical and pathogenic features, as well as novel therapeutic strategies.

## 2. Normal Skin and the Mechanisms of Cancerogenesis

Normal skin is constituted by different layers: the epidermidis, papillary, reticulum dermis and subcutaneous fat. The epidermidis is composed by four sub-layers showing different functions, including the stratum corneum that provides a barrier function and protects the other layers. In addition, melanocytes of the basal stratum exert a protective role from UV radiation. Langherans cells (LCs) play a relevant role in the activation of the immune system, while Merkel cells control the light touch. The dermis includes fibroblasts and specialized cells as well as glands, blood vessels and nerves that are variably implicated in the physiologic regulation of skin functions [22].

The mechanisms leading to the development of NMSC as well as MCC are multifactorial and include the exposition to UVR, which also represents a risk factor for both melanoma and MCC [15,23,24]. UVR determines DNA damage and the development of somatic mutations, inflammation, oxidative stress and defective activity of the immune cells. These events are milestones for the development of skin cancers. However, UVA and UVB induce different skin alterations, inasmuch as UVA promotes deeper damages, while indirectly disrupting the DNA through free radical formation, whereas UVB induces erythema, thus directly damaging DNA. Many studies have suggested that UVR is mostly adsorbed by epidermal keratinocytes and induces immune-suppression through the dimerization of cyclobutane pyrimidine, mutations in p53 and other tumor suppressor genes, as well as directly inducing inflammation and apoptosis of keratinocytes [25,26,27]. Further relevant events in the carcinogenesis process induced by UVR include free radicals-mediated damage, as well as either mutations or single-nucleotide polymorphisms (SNPs) of the glutathione S-transferase enzyme [28]. In addition, several genetic syndromes (Table 1) have been associated with both BCC and SCC and, moreover, the nevoid BCC (NBCC) shed relevant light into the pathogenic mechanisms of BCC, including the development of dedicated targeted agents. The events driving the malignant transformation of Merkel cells as result of MCPyV infection are detailed in Section 5.1.

## 3. Basal Cell Carcinoma

Basal cell carcinoma (BCC) is the most common cancer of the skin that, in as many as 80% of patients, develops in the head/neck region, often in the absence of pre-cancerous lesions. BCC rarely metastasizes, but frequently shows local invasion and tissue destruction, thus resulting in high morbidity [29]. Elderly Caucasian males, with fair skin chronically exposed to UVR, are more frequently affected in a fashion almost similar to females using sunbeds. Younger people are rarely affected, while the trunk is the most common primary site. Apart from UVR, external beam radiotherapy (RT) may also favor the occurrence of BCC, as well as arsenic, immune suppressive agents [4] and HIV infection, although a clear correlation with CD4 count has not been demonstrated. Many genetic syndromes may increase the risk of developing BCC [30] and, in this context, the nevoid BCC (NBCC) is an autosomal dominant disorder characterized by multiple lesions of the skin, pits of the palm and soles, jaw keratocysts and developmental defects [31].

### 3.1. The Genetic Landscape of BCC

A relevant topic in BCC concerns its genetic background. The deep knowledge of NBCC molecular features provides relevant information on the gene profile of BCC, thus revealing the primary involvement of the Patched 1 (PTCH1) gene. This is a transmembrane receptor that inhibits signals driven along the Hedgehog (HH) pathway [32,33,34,35]. In addition, smoothened (SMO) and glioma-associated (GLI) oncogenes have been investigated in sporadic BCC, thus revealing that loss-of-function mutations of SMO as well as alterations of the HH cascade are present in more than 90% of BCCs. The mechanisms implicated in the development of BCC are regulated by three ligands, namely Sonic- (SH), Indian- (IH) and Desert-Hedgehog (DH), whose high expression mostly occurs in the skin. They are bound by the receptor PTCH1 that, upon ligation, first activates SMO and then GLI-1, GLI-2 and GLI-3. These factors directly modulate genes implicated in tumorigenesis and angiogenesis, such as Cyclin-D1, Myc and Bcl-2. Apart from BCC, defects of the HH pathway also occur in medulloblastoma [36], breast [37], lung [38], prostate [39], colon [40] and pancreatic [41] cancers as well as lymphoproliferative disorders [42].

Unlike BCC and medulloblastoma, however, cells from solid cancers show a ligand-dependent activation of the HH pathway, although many alternative mechanisms have been described, including autocrine- (i.e., breast, lung and prostate cancer), paracrine- (i.e., colon and pancreatic cancer through both IL-6 and VEGF) and reverse paracrine-dependent HH activation, which is the consequence of soluble factors produced by stromal cells nearby tumor cells [43].

HH is critical in organogenesis, stem cell formation and tissue repair, whereas it directly controls the hair follicles and sebaceous glands of the skin [44]. Moreover, aberrant HH pathway drives a cancer stem cell phenotype, controls the primary cilium structure by triggering a complex signature that activates PTCH1 and SMO, the cytoplasmic release of GL1 proteins followed by their migration into the nucleus where upregulate genes implicated in the self-renewal, survival and angiogenesis [32,45,46,47]. The upregulation of the HH signaling is, therefore, a relevant pathogenic event occurring in more than 90% of BCC, whereas almost 10–20% of them bear SMO mutations. In addition, a non-canonical HH cascade has also been described and results mostly activated by kRAS, TGF-β and PI3K-AKT [48]. Beyond the HH pathway, other genetic alterations characterize BCC development, and they include members of the *Ras* family, *TP53*, *Hyppo-YAP* and *TERT* [43]. A “UV-signature” is frequently detected in BCC as consequence of the chronic exposition to UVA.

### 3.2. Epidemiology, Classification and Clinical Features

Data regarding the epidemiology of BCC are extremely heterogeneous, with a number of annual cases ranging from 88 to 164/100,000 persons-years across different countries. While it is possible that the real incidence of BCC is globally underestimated [8], the highest incidence rates are reported in Australia, followed by the US and Europe. The mortality of BCC is low, and it is mostly influenced by concurrent diseases, age and clinical complications, whereas it occasionally depends on extensive tissue infiltration and metastatic spreading that include either nodal or distant site involvement [49]. Development of advanced BCC mostly occurs in males and is associated with worse prognosis and younger age [50,51,52,53,54].

**Figure 1:**
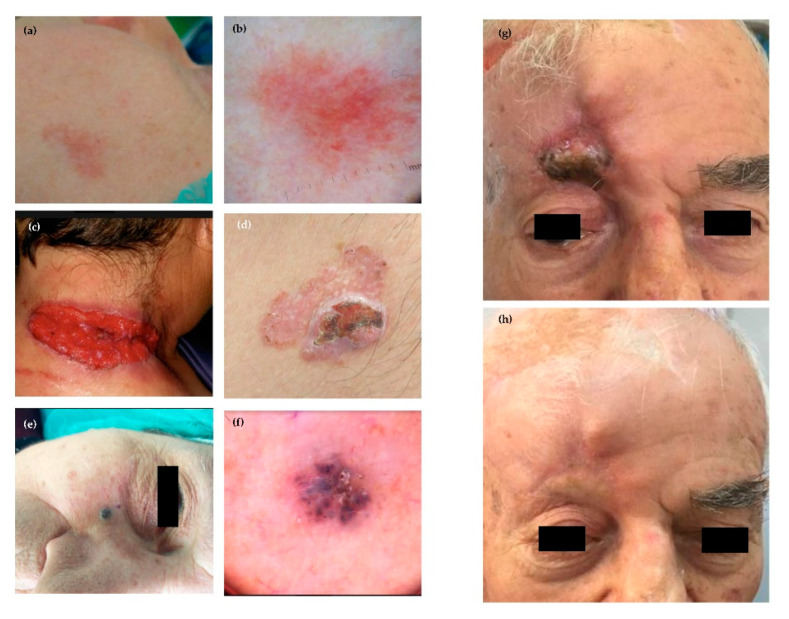
Representative clinical patterns and dermatoscopy of BCCs: (**a**) clinical features and (**b**) dermatoscopy of superficial BCC of the cheek; (**c**) ulcerated and (**d**) multifocal BCC; (**e**) nodular pigmented BCC of the zigomatic area and (**f**) relative pattern by dermatoscopy; and (**g**,**h**) Effect of Hedgehog inhibitors in a patient with advanced BCC of the head.

**Figure 2:**
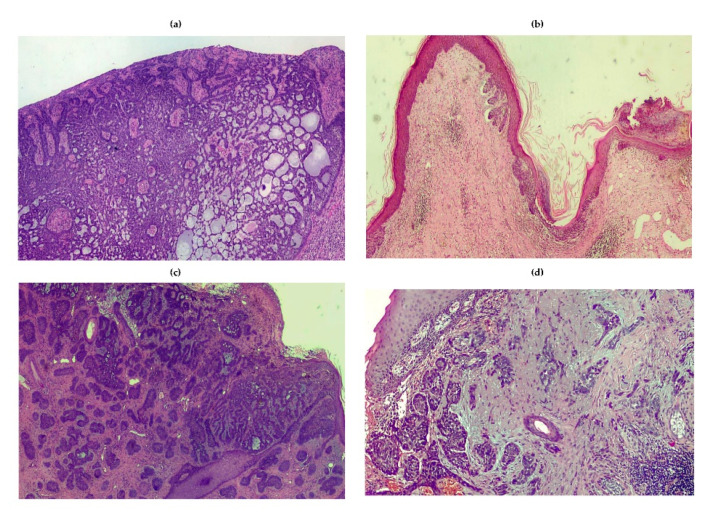
Histologic patterns of BCC: (**a**) adenoid variant of nodular BCC showing island of tumor cells characterized by a cribriform pattern; (**b**) superficial BCC; (**c**) micronodular BCC; and (**d**) morpheaform variant showing malignant cells surrounded by a sclerotic stroma enriched in collagen. The infiltrative features are also shown.

The clinical features of BCCs are extremely heterogeneous (Figure 1), and a universal classification is currently unavailable [55,56]. Clinical variants can be subdivided into: (i) nodular; (ii) superficial; (iii) dibroepithelial; and (iv) morpheaform. Some BCCs contain melanin, while nodular pattern may characterize any histologic variant. The nodular BCC shows high propensity to ulceration, as well as worse prognosis. Other variants include the cystic, mucinous, basosquamous and micronodular as well as multifocal BCC (Figure 2). In particular, the basosquamous BCC is a mixed variant characterized by histologic features of both BCC and SCC, showing high aggressiveness including its capability of local and distant metastasis [18].

#### 3.2.1. Nodular

It is the most common variant accounting for 50–79% of BCCs [57]. Lesions are mainly characterized by a papule or a pearly nodule. The nodular BCC is often ulcerated and pigmented, or it shows a central depression and is frequently bleeding. The head/neck is the most common primary site.

#### 3.2.2. Superficial

It is the second commonest clinical subtype [58]. Its prevalent feature is the appearance as macula, atrophic plaque, papula or erythema-like lesion that rarely results pigmented, well-defined, scaly and pinkish. Regression is a common feature of this type of BCC. The trunk and extremities of younger people as well as head/neck district are the most frequent primary sites. Multiple superficial BCCs may occur. The majority of superficial BCCs show a horizontal pattern of growth, rather than a vertical one, whereas ulceration, nodular features and invasive pattern are rarely observed. Notwithstanding a number of histologic variants and rare patterns, they have no relevant prognostic implications, apart from a modest propensity to local diffusion and distant metastasis [58].

#### 3.2.3. Fibroepithelial

It is a rare form that mostly involves the trunk, mainly occurring as a pink-colored plaque, sessile or papula-like lesion [57]. It may include pigment.

#### 3.2.4. Morpheaform

This is a rare variant of BCC (5–10%) characterized by an elevated or depressed pink/ivory and indurated plaque showing a smooth surface that often includes telangiectasias [59]. This form of BCC is highly aggressive with an elevated attitude to local invasion and distant metastasis.

#### 3.2.5. Infiltrative

This variant is similar to morpheaform BCC and is mostly characterized by a heavy stromal fibrosis with dense collagen bundles; it grows in a poorly circumscribed fashion and often invades the subcutis, while tumor cells spread forming a large irregular nodule [59].

#### 3.2.6. Micronodular

This form resembles the classical nodular BCC and seems characterized by a deep extension into the dermis, as well as sporadic infiltration of the subcutis with stromal proliferation [60].

#### 3.2.7. Basosquamous

It shows infiltrating jagged clumps of tumor cells, some with a clear-cut basaloid morphology and cytoplasmic keratinization [61].

### 3.3. Therapeutic Options

The therapeutic strategy of BCC should be based on a multidisciplinary approach, although surgery (either curative or palliative) remains the primary option. Surgery requires a skin cancer board of experts and finality includes type of excision, adequate margins, appropriate techniques of reconstruction, tissue preservation and dedicated surgical approaches in certain difficult sites that require a topographic study of the of primary tumor as well as the early planning of adjuvant options that include both systemic therapy and RT [3].

#### 3.3.1. Radiotherapy

RT should be regarded as the primary treatment in patients bearing BCC and considered not eligible for surgery due to advanced disease stage, comorbidities, risk of complications and location of primary sites (i.e., eyelid, nose or lip). A systematic review [62] reported an estimated recurrence rate of 3.5% after RT, with data similar to both surgery and micrographic technique developed by Mohs [63]. However, the principal indications for RT include: (i) inoperable tumors; (ii) the certainty of disfigurement that is not balanced by the certainty of clear margins; and (iii) incomplete resection with microscopic or macroscopic residual tumors [64].

Finally, external beam RT remains the most used treatment, although other options include brachytherapy that provides similar results in terms of recurrence-free survival and local complications [56,65].

#### 3.3.2. Systemic Treatments

Early treatment of BCC was mainly based on the use of systemic or topic Imiquimod as well as 5-fluorouracil-based chemotherapeutic agent that unfortunately produced only modest improvements in terms of median OS [66]. Although it still represents an indication in a small group of patients, the knowledge of the mechanisms activated along the HH pathway has allowed developing and testing several targeted therapies. They include vismodegib, a small molecule optimized to inhibit SMO, and additional compounds including sonidegib, itraconazole and others at different stage of development. Both Vismodegib and Sonidegib have been approved for the treatment of locally advanced or metastatic disease, as well as for patients not candidate to surgery or radiotherapy [67,68,69,70]. In addition, itraconazole has shown efficacy and manageable safety in an exploratory phase 2 study (NCT01108094), while patidegib, glasdegib and talasdegib (LY2940680) are promising drugs under investigation that, by blocking HH-mediated signals, are indicated for the treatment of advanced disease [71,72]. The majority of HH inhibitors have proved able to improve OS and delay the recurrence in a high number of patients, while showing an acceptable safety profile, as demonstrated by the STEVIE clinical trial [73].

However, patients receiving HH inhibitors commonly experience adverse events of any grade that include alopecia, muscle spasms, fatigue, vomiting and dysgeusia, not taking into account the development of resistance [68,74]. Thus, intermittent, or dose escalation, schedules allow overcoming these complications; notably, Grade 3–4 toxicities causing definite treatment breaks are frequently associated with a poorer outcome [75,76]. Notwithstanding the experience from the real-life treated population is extremely puzzling, relevant results concerning clinical predictors of response have emerged from pivotal trials. In particular, the best response proved to be achieved in locally advanced disease, younger people, tumors smaller than 4 cm and absence of prior exposure to other HH inhibitors, whereas histology apparently does not influence outcome. Weekly interval dosing ameliorates adverse events, but a prolonged drug discontinuation has been associated with tumor progression. Moreover, intermittent schedules have been explored in the MIKIE phase 2 trial, which produced inconclusive results [76]. To date, there are no comparative studies analyzing potential difference between Vismodegib and Sonidegib, although indirect comparisons have been conducted using experimental trials’ results. The result of both the ERIVANCE [68] and BOLT trials [70,77] were similar, although Vismodegib showed an apparent advantage in the metastatic setting.

Re-treatment is an effective challenge in the majority of patients, whereas new indications are emerging, including neo-adjuvant treatment for locally advanced BCC, to be pursued until clinical response, followed by definitive surgery. This strategy is indicated for difficult lesions mostly located in peri-ocular and orbital areas.

#### 3.3.3. Innovative Therapeutic Strategies

Based on the variability of response mostly due to the heterogeneous clinical presentation of these malignancies, recent data completed in other cancer models (e.g., medulloblastoma) show a high responsiveness to HH inhibitors, revealed a predictive gene profiling represented by deregulated genes such as *GLI1*, *SPHK1*, *SHROOM2*, *PDL1M3* and *OXT2* [3,74]. In addition, phase 1 studies demonstrated that *GLI1* levels are decreased by treatment, and thus suggested its expression as a pharmacodynamics marker of treatment [75]. The BOLT trial [70], however, extended this observation to BCC, thus suggesting *GLI1* as candidate master gene in BCC. Other biomarkers validated in pre-clinical models and phase 1 studies are *MSI2*, *CCND2*, *PITCH1* and *BAFF,* as well as macrophage inflammatory protein-1 alpha, IL-8 and CCL19 deregulated expression [78].

Another interesting issue in the management of BCC concerns the mechanisms implicated in the acquired resistance. In this context, acquired mutations in *SMO* cause resistance, as well as amplification of downstream genes such as *GLI2* and mutations of genes implicated in the control of ciliogenesis such as *CFD1* and *SNFN,* which apparently promote resistance through SMO-independent pathways [79,80]. A relevant approach for overcoming resistance is to target different downstream mediators such as *DYRK1B,* whose antagonism is of great effort in decreasing *GLI* levels and thus restoring the sensitivity of malignant cells [81]. Furthermore, another strategy includes the use of mTOR inhibitors [79], itraconazole [82] or anti-PD1 monoclonal antibodies, which showed promising anti-proliferative activity in patients progressing after an HH inhibitor.

## 4. Squamous Cell Carcinoma

Squamous cell carcinoma (SCC) is the second most common skin cancer that develops more frequently in Caucasian subjects exposed to environmental factors, as well as UVR, smoking, chronic infections and immune suppressors, or bearing peculiar genetic background. Detailed epidemiologic data are not available worldwide, and the incidence in the next decade in Europe is expected to approximately double, although the most reliable epidemiological information are collected in Australia, USA, and the Swedish Cancer Registry [83]. Mortality is correlated with the ability of malignant cells to spread toward distant sites, as well as with older age, male gender, site (i.e., lip, temple and ear), thickness, transplantation, treatment with BRAF inhibitors, HIV infection or chronic lymphatic leukemia (Table 2). Many studies have been published dealing with the increased risk of SCC following treatment with BRAF inhibitors in metastatic melanoma patients. These studies have clearly demonstrated that this targeted therapy induces a hyper-proliferation of keratinocytes, due to paradoxical activation of the mitogen-activated protein kinase (MAPK) pathway, in BRAF wild-type cells. This event mostly occurs in patients bearing oncogenic mutations of Ras and is, at least in part, reverted by the combination with MEK inhibitors [84].

### 4.1. The Molecular Features of SCC

Studies on the genetic landscape of SCC demonstrated that genes altered by UVR exposition are *TP53*, *CDKN2A*, *NOTCH1*, *NOTHC2*, and *p16* suppressor gene; epigenetic regulators such as *KMTC2, KMT2A, ARID2, SETD2, CREBBP* and *TET2*; mutations of *TGF-β receptor*; and mutations in DNA repair pathways including missense mutations in *ATR*, *PIK3CA, ERRB4* and *NF1* [85,86,87]. In the context of epigenetic modifications, a number of microRNAs (miRNAs) proved to be upregulated or downregulated in SCCs, and those exhibiting oncogenic functions (miRNA-21, -205, -181a, -125b, -34a, -148a, -214, -124 and -199a) have been found modified with respect to normal skin tissues. Thus, potential therapeutic strategies by antisense oligonucleotides are under investigation in pre-clinical studies [88,89]. In addition, such targeted therapies may induce PI3K and EGFR defects while chronic exposure to azathioprine has been associated with a peculiar hyperactivity of endogenous cytidine deaminases (APOBEC) in SCC developed in consequence of recessive epidermolysis bullosa [90]. Other studies discovered a number of SNPs associated with SCC as well as transcription factors and metastasis suppressor genes (i.e., CADM1 and AHR). Lastly, the microenvironment has a key role in enhancing or blocking the proliferative extent of cancer cells, and a major effect is played by HLA variants and PD1/PDL-1 interplay.

Finally, SCC has been found associated to hereditary syndromes as xeroderma pigmentosum, epidermolysis bullosa, oculocutaneous albinism, Fanconi anemia and Lynch syndrome [91,92].

### 4.2. The Clinical Characteristics of SCC

**Figure 3:**
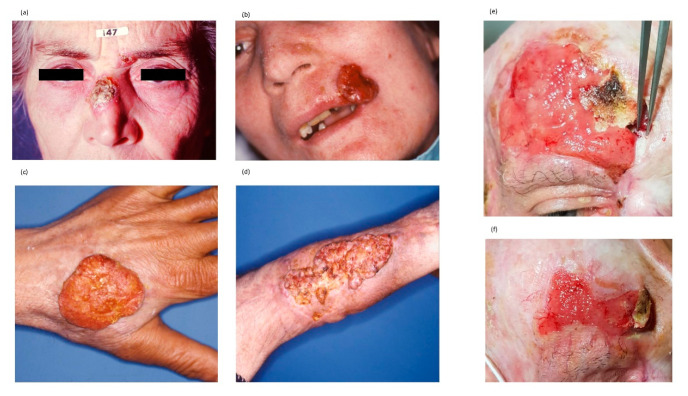
Clinical features of SSCs. Panels are representative of clinical presentation and response to immunotherapy: (**a**) SCC of the nose that arises on photo-damaged skin in presence of actinic keratosis of the left eyebrow; (**b**) high grade SCC of the left commissura of the lower lip; (**c**) SCC of the hand in patients in active treatment for concomitant LLC; (**d**) SCC originated on previous burned skin; and (**e**,**f**) effect of six-months course of anti-PD-1 MoAb in a patient with locally advanced SCC unfit for further surgery.

**Figure 4:**
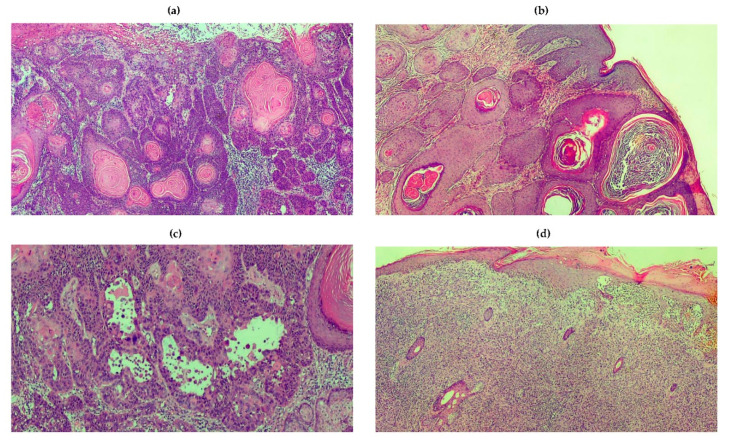
Histologic variants of SSC. Panels are representative of histologic patterns from patients with SCC. (**a**) A moderately differentiated and ulcerated lesion showing enlarged, hyperchromatic and irregular nuclei. Corneal pearls in the middle reflect the keratinization ability. Malignant cells are surrounded by abundant inflammatory cells. (**b**) Verrucous SCC characterized by deeply invasive properties. (**c**) Spindle cell SCC showing elongate, fusiform cells that blend with the surrounding reactive fibroblastic component. (**d**) Acantholytic SCC characterized by a pseudoghiandolar pattern and dyskeratosis of tumor cells. The acantholytic phenomenon affects the inner portion of invasive nests and lobules.

SCC includes many different subtypes, endowed by different clinical features ranging from an indolent behavior with slow growth to aggressive tumors showing invasive properties and high spreading toward distant sites (Figure 3) [93,94]. In patients receiving appropriate treatments, the risk of recurrence is about of 5%, while nodal and distant metastases occur in 4–6% of patients with a variability that almost depends on the histologic pattern and risk factors (Table 2). The clinical presentation is extremely heterogeneous in relation to the site, size, thickness and pigmentation. There is no accepted consensus on SCC classification, but superficial SCC is considered the most common variant that often develops from an actinic keratosis (AK) or a Bowen disease, although the risk of malignant transformation of these diseases is overall extremely low (Figure 4). However, the last edition of the WHO classification includes new variants including those SCC which develop from a cheratoacanthoma [84,95,96,97]. Notwithstanding, the majority of SCC are characterized by keratinocyte dysplasia that progressively involves the epidermis and derma, as well as the surrounding stromal tissues; furthermore, SCC may already be invasive at diagnosis. The majority of metastatic SCCs originate in the head and neck district, as well as in sun-exposed skin. In relation to grading, SCCs are classified into G1, G2 and G3.

Many SSCs develop as a plaque or a papule with a hyperkeratotic surface, often pigmented [96]. Based on the differentiation grade, the primary lesion may be verrucous, seborrhoic keratosis, crateriform, brown or light. Otherwise, it is ulcerated and red-fleshy non-keratotic lesion that in most instances may resemble an amelanotic melanoma, a cutaneous metastasis or a MCC. Poorly differentiated SSCs are larger tumors with induration, which may also involve surrounding skin and high propensity to infiltrate nearby structures. Metastatic SCCs are characterized by in-transit, nodal and visceral metastases. The majority of SCCs are diagnosed by means of a simple clinical examination, although dermatoscopy may be conclusive in the case of difficult patterns. Other non-invasive diagnostic techniques include in vivo reflectance confocal microscopy and optical coherence tomography, but their effective application in the clinical practice needs further confirmation.

### 4.3. The Immune System and SCC

The balance between cancer and immune system is a milestone for the malignant cell development and this event is considered critical for the SCC development [98,99]. Many studies have suggested that disruption of immune-editing phases is required for SCC, while both UV exposure and viral infections cooperate for the impairment of immune system response. Both innate and adaptive immunity apparently play a role in tumor antigen clearance in SCC [100] and small subsets of clonal cells are reprogrammed to favor immune suppression in favor of tumor cell proliferation [89]. The development of SCC is mostly based on a tight interplay between tumor and immune cells that leads to a favorable microenvironment [101,102]. It has been demonstrated that UV promote chronic inflammation that surrounds malignant squamous cells, by recruiting macrophages, and promoting both apoptosis and defective lymph node migration of specialized cutaneous dendritic cells (DCs), namely Langherans cells (LCs) as well as monocyte-derived dermal DCs [103,104,105]. Other immune populations actively deregulated in SCC development are natural killer cells (NKs) and innate lymphoid cells (ILCs) that are key regulators of Th1, Th2 and Th17 immune response. By contrast, modest data are known about myeloid-derived suppressor cells while two subsets of T-cells, namely y∂ and NK-T cells, apparently exert a major role. In this context, the dendritic epidermal y∂ T-cells (DETCs) are the best studied population. Moreover, the tumor microenvironment is engulfed of immune suppressive cytokines such as IL-6 and IL-10 as well as T-regulatory cells whose migration nearby tumor cells contribute to enhance the evasion from the immune system control. A recent mechanism described in SCC and activated by UVR exposition concerns specific photoreceptors such as urocanic acid, a molecule that is found at high concentration in the corneum stratum of the skin. The isomerization of the urocanic acid leads to the development of its cis-form that is critically involved in the transient alteration of the immune surveillance, thus favoring the immune system impairment. In addition, it has been demonstrated that urocanic acid directly interferes with T-cells, thus promoting the expansion of immune suppressive subsets [106].

The acquired immune response in SCC is at least in part dependent on the ability of T-cells to drive a Th1- or Th2- and Th17-mediated immune response. It has been demonstrated that tumor-specific T-cell response is a pre-requisite for preventing skin cancer, while IL-22 overproduction is a hallmark of high keratinocyte turnover. In addition, the SCC scenario is also influenced by the quality of B-cell response that may restrain the immune response through TNF-α and IL-10 overproduction, whereas a defective number of infiltrating CD20⁺ cells has been also demonstrated in humans. In this context, several cytokines and signaling molecules are variably involved in the immune evasion that characterizes SCC, and a peculiar association of IL-10 haplotypes, IL-4R and TNFR2 with an increased risk to develop SCC has clearly been demonstrated [107].

The interplay between malignant cells and surrounding stromal cells is a milestone for understanding the role of microenvironment in tumor progression [108]. In this context, an active network is regulated by signals driven by cytokines, chemokines, growth factors, inflammatory cells, immune cells and enzymes involved in stroma remodeling [109,110,111,112,113,114,115,116]. Moreover, the characteristics of tumor-infiltrating lymphocytes reflect the effective anti-cancer role of the immune system. In addition, the deep understanding of the tumor microenvironment has suggested that many somatic mutations in SCC acting as neo-antigens may be targeted by cytotoxic cells. Data from the early studies in melanoma using an anti-CTLA4 MoAb show that SCC is characterized by a mutational load of around 50 mutations per megabase of DNA. In addition, PD-1 expression was widely demonstrated on T-cells surrounding tumor cells that, conversely, showed a PD-1 level of about 30%. High PD-1 expression in the context of a “cold” microenvironment was associated, however, with an increased risk of metastases and was also identified in lymphatic disease, thus providing definitive evidence for the prognostic role of immune infiltration in advanced SCC. In this context, recent data highlight the role of mutational landscape and HLA haplotypes in modulating the pressure of the immunological profile of cancer cells, thus potentially favoring response to immunotherapy [89,92,117,118].

### 4.4. Systemic Treatments

The gold standard therapy for SCC is definitely surgery, with alternative options including laser dissection, intra-lesion drug injection and electrodissection. However, other strategies have been considered in those patients considered unfit for surgery in relation to comorbidities, site of primary tumor, risk of local infiltration or quality of curative margins. They include external beam radiotherapy (RT) and brachytherapy. Retrospective analyses revealed a high number of biases in RT-based studies, mostly due to the heterogeneity of randomization, although an improvement in terms of disease-specific survival and OS has emerged in the majority of studies. In addition, there are no reliable data for other adjuvant treatments of SCC and, therefore, the indication for RT include lesions of the head/neck with regional node metastases and extracapsular extension, patients with positive margins or those not candidate to surgery due to concurrent diseases or difficult primary sites [96].

The poor results obtained by conventional chemotherapy in patients with advanced SCC and the results of original studies showing the high number of somatic mutations and neo-antigen load suggested to plan clinical trials with immune checkpoint agents (mainly blocking PD-1) in patients excluded from other strategies. In a fashion almost similar to melanoma, PD-L1 levels did not correlate with clinical response to anti-PD1 mAbs). Therefore, the immune checkpoint inhibitors cemiplimab and pembrolizumab have been approved in US for the treatment of locally advanced or metastatic SCC [119,120,121]. Based on these data, other clinical phase 2 studies with neoadjuvant Cemiplimab in stage III–IV SCC of the head/neck demonstrated its potential benefit in preliminary studies. The R2810-ONC-1540 was a phase 2, open-label study accruing 193 patients with locally advanced or metastatic SCC (NCT02760498). The study demonstrated an overall response rate (ORR) of 49.2% with 17% complete responses, 32% partial responses and 15% stable diseases, whereas 17% developed progression that mainly occurred in the metastatic group (27%).

Apart from chemotherapy, other strategies are based on the high EGFR expression demonstrated in SCC; indeed, its levels correlated with outcomes. Thus, anti-EGFR MoAbs combined with chemotherapy or RT is considered an option in patients showing progression after first-line regimen with anti-PD1 agents. Currently, systemic chemotherapy has not been approved for SSC based on the modest results in terms of response, coupled with the cost of serious adverse events, especially in a fragile patient population. On the contrary, a relevant disease control and local response has been obtained by electrochemotherapy; indeed, the EURECA trial investigated this option in skin cancers achieving a 55% of response in SCC [122]. Several patients developing SCC are immune depressed by transplantation, whereas others are affected by HIV-1 infection or hematological disorders, and thus are often excluded from immunotherapy trials. In this context, adoptive cell transfer using chimeric antigen receptor T-cells (CAR-T cells) seems to represent a novel alternative strategy to be potentially pursued in this niche of patients [123,124,125].

## 5. Merkel Cell Carcinoma

Merkel cell carcinoma (MCC), also known as primary cutaneous neuroendocrine carcinoma, is a rare malignancy of the skin characterized by an aggressive clinical behavior [23]. Although MCC is rare, its incidence is rising steadily, probably both as a result of improvements in diagnosis, as well of the global ageing of the world population. According to data from the Surveillance, Epidemiology, and End Results (SEER) database, the incidence of MCC increased from 0.5/100,000 individuals in 2000 to 0.7/100,000 persons in 2013 [126], and similar trends have been reported across Europe and Australia [127,128,129]. The incidence of MCC progressively increases with every additional decade of life, and only 4% of MCC cases occur in patients under 50 years of age [130]. The malignancy predominantly affects subjects of white ethnicity, and its frequency is higher in geographic areas closer to the equator, thus suggesting an association between UV radiation and disease occurrence [131]. Furthermore, transplant recipients and patients with B cell malignancies have an increased risk of developing MCC. In particular, the standardized incidence ratio of MCC in patients with chronic lymphocytic leukemia has been estimated to be 15.7 (95% CI, 3.2–46) [132].

### 5.1. Pathogenesis of MCC

MCC is a poorly differentiated neuroendocrine carcinoma that lacks a recognized benign or dysplastic precursor. Traditionally thought to arise from Merkel cells, MCC more likely derives from a yet to be defined cellular population which underwent neuroendocrine differentiation before or during malignant transformation. Pro-B lymphocytes, pre-B lymphocytes, fibroblasts, dermal mesenchymal stem cells and epidermal progenitor cells are among the most investigated candidates as cell of origin, but the possibility that MCC may originate from multiple, distinct, cells cannot be ruled out at present [15]. Both viral and non-viral factors play a key role in the pathogenesis of MCC. Merkel cell polyomavirus is the causative agent of a substantial fraction of MCC cases. It was discovered in 2008 as a new member of the Polyomaviridae family of small, non-enveloped, double-stranded DNA viruses, and five geographically-related genotypic variants have been characterized thus far [133]. MCPyV is part of the human skin microbiome, being chronically shed from infected cells in the form of assembled virions. The virus determines asymptomatic infections of the skin and is highly prevalent in the population, with anti-MCPyV antibodies detected in as many as 50% of children and 80% of older individuals [134]. MCPyV-related oncogenesis follows a model of multi-step progression, in which a sequence of distinct events is required to induce the malignant transformation. First, the MCPyV genome is linearized and integrated into the host genome after a concurrent DNA-damaging event, such as UV exposure. Second, infected cells are forced to express two viral oncoproteins, namely small tumor antigen (sT) and large tumor antigen (LT). While sT has oncogenic activity per se, by inhibiting the proteasomal degradation of cyclin E and c-Myc, LT acquires pro-tumorigenic activity only when mutations of the 3′ end of the gene lead to the loss of the protein C-terminus. Indeed, truncated LT inactivates the tumor suppressor Rb, driving uncontrolled cell proliferation. Following tumor formation, multiple mechanisms contribute to cancer cell survival in the presence of a destructive immune response. In addition, MCPyV-specific T cell responses have been detected both locally and systemically in patients with MCC, but the frequent expression of PD-L1 by cancer cells disables their effects by inducing T cell exhaustion [15,135,136,137,138,139]. In this context, the defective expression of HLA class-I by tumor cells may hamper antigen presentation, further promoting immune evasion [140].

While the majority of MCC cases recorded across US and Europe are virus-positive, up to 80% of tumors diagnosed in Australia have negligible levels of MCPyV-associated antigens [141,142,143]. The mechanisms underlying the pathogenesis of MCPyV-negative MCC still need to be completely elucidated, but the observation that virus-negative MCCs are characterized by UV mutational signature supports the idea that UVR might play a pivotal role in the development of the neoplasm [144], which is definitely credible in a country such as Australia. Notably, virus-negative MCCs have a substantially higher mutational burden as compared with virus-positive tumors and harbor recurrent, clonal, inactivating mutations of *TP53*, *RB1* and other genes involved in the Notch signaling that are not frequently observed in the MCPyV-positive counterpart [145,146,147,148].

### 5.2. The Clinical Presentation and Diagnosis of MCC

MCC typically presents as a solitary, painless, red or violaceous intracutaneous nodule rapidly growing on the sun-exposed skin of elderly, fair-skinned individuals. In an analysis of 9387 cases recorded in the US National Cancer Database between 1998 and 2012, MCC was diagnosed at local, locoregional, or metastatic stage in 65%, 26% and 8% of cases, respectively [149]. Skin, lungs, adrenals, liver, brain and skeleton are the preferred sites of metastasis. Nevertheless, in up to 15% of patients, lymph-node involvement is detected in the absence of a recognizable cutaneous tumor, possibly as result of the spontaneous regression of the primary tumor [150,151]. MCC regression is associated with improved prognosis [152], but little is known regarding the biological mechanisms leading to the disappearance of the primary tumor.

**Figure 5:**
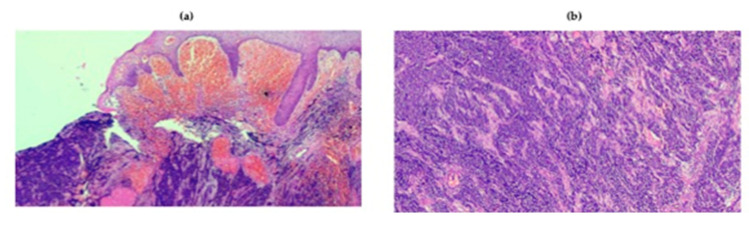
Representative histologic patterns from MCC. (**a**,**b**) Histology of MCC characterized by deep infiltration of the dermis by lymphocytes at 4× (**a**) and 10× (**b**) magnification.

The diagnosis of MCC is clinically challenging, thereby relying almost entirely on histology examination. Morphologically, MCC is characterized by the aggregation of small, monomorphic, round cells with scant cytoplasm in the context of nodules or sheets located in the dermis or subcutaneous tissue (Figure 5) [151]. Metastatic small-cell lung cancer (SCLC), small-cell melanoma, Ewing’s sarcoma and some lymphomas can have pathological features similar to those of MCC, and immunohistochemistry (IHC) appears useful in the differential diagnosis of these entities. Classical IHC markers of MCC include chromogranin-A, synaptophysin, cytokeratin 20 (CK20) and MCPyV-associated antigens. The negativity of thyroid transcription factor 1 (TTF1) may enable the distinction between MCC and SCLC metastatic to the skin, while the assessment of the Ewing’s translocation may be necessary to rule out a cutaneous metastasis of Ewing’s sarcoma. Strikingly, MCC and SCC may co-exist in the same lesion, possibly as UV-induced unrelated malignancies or, intriguingly, as tumors originating from the same multi-potent stem cell, and diverging in their differentiation at a later stage [151].

Following a pathological diagnosis of MCC, an accurate staging of the patient is mandatory. Both the American Joint Committee on Cancer (AJCC) and the Union for International Cancer Control (UICC) recommend the use of the eighth edition of the TNM system in routine clinical practice [153]. Given the higher sensitivity shown by ^18^FDG-PET/CT imaging with respect to CT or MRI, functional imaging is presently considered the gold-standard procedure for the clinical assessment of MCC at diagnosis and follow-up [154]. In patients with radically resected MCC, the risk of recurrence is especially high within the first two years from the original diagnosis, and surveillance imaging should thus be performed every 3–6 months in this timeframe. The titers of antibodies against MCPyV T antigens have been shown to correlate with disease burden, and their increase is associated with tumor recurrence or progression, thus providing a non-invasive tool for follow-up individualization [155,156].

### 5.3. The Role of Sentinel Lymph Node Biopsy and of Systemic Treatments

The management of MCC patients primarily depends on the disease stage at presentation. In subjects without evidence of lymph-node involvement, sentinel lymph node biopsy (SLNB) is generally indicated. Patients with negative SLNB should undergo surgical excision with 1–2 cm margins, or definitive RT if surgery is not technically feasible [157]. By contrast, when the SLNB is positive, a careful assessment of occult metastatic disease should be carried out, and patients should be treated systemically if in stage IV, or with definitive surgery, RT or a sequence of surgery and RT in presence of regional lymph-node involvement [157]. Adjuvant RT can be recommended for patients with local MCC at high risk of relapse, including those who did not undergo SLNB [157,158,159]. While the role of adjuvant chemotherapy is highly debated, multiple trials are currently investigating immune checkpoint inhibitors, including pembrolizumab (NCT03712605), nivolumab (NCT03798639), ipilimumab (NCT03798639) or avelumab (NCT03271372), in the adjuvant setting and their results are awaited soon.

In this context, the phase 1/2 CheckMate 358 study [160] has recently tested nivolumab for the neoadjuvant treatment of 39 patients with stage IIA/IV, resectable MCC. Among the 36 patients who underwent surgery, 17 (46%) achieved a pathologic complete response, with tumor shrinkage being observed irrespective of MCP V detection, PD-L1 expression or tumor mutational burden. After a median follow-up of 20 months, both median recurrence-free survival and OS were not reached, in the presence of Grade 3/4 adverse events in just 8% of the whole cohort of patients. Notably, adverse events and tumor progression hindered the surgical intervention in two and one enrolled patients, respectively, thus emphasizing the importance of treatment tailoring in these subjects. Systemic therapeutic options for patients with stage IV are represented by either chemotherapy or immunotherapy.

Chemotherapeutic regimens including platinum-based combinations, etoposide, topotecan, taxanes and anthracyclines were widely used until 2016, leading to response rates in the range of 30–75%, and to median PFS and OS of approximately 3 and 10 months, respectively, in the first-line setting [161,162,163,164,165]. The immunosuppressive effect of chemotherapy is currently regarded as a possible mechanism accounting for the early development of resistance following treatment with cytotoxic agents in the context of a highly immunogenic cancer [166]. Therefore, cytotoxic chemotherapy is presently reserved to patients who are not candidates to immunotherapy (i.e., organ transplant recipients or patients with autoimmune diseases) or who have progressed to immune checkpoint inhibitors. The PD-1/PD-L1 axis is a key therapeutic target in MCC, and immunotherapy is currently recommended as the preferred first-line option in patients with advanced disease. Avelumab is a fully human IgG1 mAb directed against PD-L1, and its safety and efficacy have recently been investigated in the phase 2 JAVELIN Merkel 200 trial [167]. In Part A of this study, 88 patients with MCC progressive to at least one line of chemotherapy received avelumab at 10 mg/kg every two weeks. After a median follow-up of 29 months, objective responses were documented in 33% of cases, in the presence of 11% complete response rate. Strikingly, responses were durable, with 67% of them lasting over two years. The two-year PFS and OS rates were 26% and 36%, respectively. Notably, no substantial differences in terms of antitumor activity were seen between MCPyV-positive and -negative tumors, as well as between PD-L1-positive and -negative MCCs [168]. Part B of the JAVELIN Merkel 200 trial, aimed at investigating the safety and efficacy of first-line avelumab monotherapy in MCC, has recently completed the accrual of 112 patients. In a pre-planned interim analysis of 29 patients with at least three months of follow-up, the objective response rate was 62%, with a duration of response exceeding six months in the 83% of responding patients [169]. No Grade 4 toxicities have been reported in the JAVELIN trial, while Grade 3 adverse events have been documented in only 5% of patients enrolled in Part A of the study. In an analysis of 240 patients with advanced MCC receiving avelumab in the context of the expanded access program of the JAVELIN trial, the PD-L1 inhibitor confirmed a manageable safety profile [170]. Both FDA and EMA have approved avelumab for the treatment of adult patients with metastatic MCC.

The PD-1 blockers pembrolizumab and nivolumab have been recently tested in patients with advanced MCC. In a phase 2 study enrolling 50 patients with metastatic or recurrent locoregional MCC naïve to systemic therapy, pembrolizumab at 2 mg/kg every 21 days determined objective responses in 56% of cases, with a complete response rate of 24%. After a median follow-up of 14.9 months, the median PFS was 16.8 months, while the two-year OS rate was 69%. Again, no differences were detected according to either MCPyV or PD-L1 status in terms of response rate or duration of response. Grade 3 or greater adverse events were reported in 28% of patients, leading to treatment discontinuation in 14% of cases [171,172]. On this basis, the FDA granted accelerated approval to pembrolizumab for patients with recurrent locally advanced or metastatic MCC. Finally, the phase 1/2 CheckMate 358 trial has recently tested nivolumab at 240 mg every 14 days in 25 patients with advanced MCC. Among 22 evaluable patients, the overall response rate was 68%, with responses occurring in both treatment-naïve and pretreated patients, irrespective of the viral and PD-L1 status.

Innovative immunotherapeutic approaches for the treatment of advanced MCC patients include combinations of immune checkpoint inhibitors (including MoAbs against CTLA4 and LAG3), adoptive T cell or NK cell immunotherapies, as well as oncolytic viruses such as talimogene laherparepvec [139,173].

## 6. Conclusions and Future Perspectives

The progress in understanding the pathogenesis of BCC, SCC and MCC has allowed developing novel therapies, thus leading to great impact on survival and quality of life in many patients. To this regard, the knowledge of the genetic landscape of BCC has definitely proved that the cascade of signals driven through the HH pathway is crucial for the proliferation of cancer cells, and its inhibition by dedicated targeted agents restrains this property. However, other genetic defects have been discovered and new agents suggested for the treatment of this type of cancer, including blockers of PD-1 and PD-L1 signals. In this context, the immune system plays a key role in SCC pathogenesis and pre-clinical models have provided critical insights about alterations of immune cells that regulate the skin cancer biology. However, there is unlikely to be a single trigger for SCC development because the combination of genetic and environmental factors is of great effort for the malignant transformation of keratinocytes. Moreover, the comparison of premalignant with malignant skin tissues has permitted to reveal proteomic, genomic and immunological differences associated with cancer development. Other studies focused on microenvironment defects in NMSC, suggesting that dynamic interplay exists between malignant cells and those regulating either innate or adaptive immune system. The knowledge of these events has progressively changed the landscape of metastatic SCC treatment, thus providing novel options that are also under investigation in MCC and BCC. However, further information regarding the neoantigen load, the characteristics of the immune infiltrate and cells of the microenvironment are required to optimize the immunotherapy in NMSC. In this context, new techniques are under investigation to overcome actual limitations, with the purpose to tailor the treatment in relation to the continuous phenotypic and antigenic modifications that characterize cancer cells.

## Figures and Tables

**Table 1:** Genetic syndromes associated with NMSC.

Syndrome	Type of NMSC	Clinical Features
Xeroderma Pigmentosus	BCC SCC	Autosomal recessive disorder characterized by defects in the mechanisms of DNA repair. NMSCs are frequently developed by younger (≤20 years). The risk is directly associated to UVR exposition.
Oculocutaneous Albinism	SCC	Autosomal recessive disease showing a pigmentary dilution of the skin, eyes, and hair. SCCs are frequently developed by young subjects exposed to UVR. A high incidence of metastatic SCC has been described in black population.
Epidermodysplasia Verruciformis	SCC	Rare and usually recessively inherited disorder characterized by a colonization of the skin by HPV. The majority of patients develop NMSC as adults, usually in sun-exposed regions, but earlier with respect to the general population. Aggressive biological behavior includes perineural spread, metastases and death.
Dystrophic epidermolysis bullosa	SCC	It is characterized by both dominant and recessive mutations of the type VII collagen gene. The majority of recessive patients develop SCCs, that occur during the third-fifth decade of life, frequently multiple and with high attitude to local recurrence and spreading to distant metastatic sites.
Basal Cell Nevous Syndrome (Gorlin)	BCC	It is an autosomal dominant disorder caused by inactivating mutations of *PTCH1* or rarely *PTCH2*. Clinical features include early NMSC, involvement of multiple sites on course of life, odontogenic keratocysts of the jaw, palmo-plantar pits calcifications of the falx cerebri and abnormalities of the skeleton. Patients also develop multiple cancers of the SNC, ovary and heart, as well as other skin defects such as epidermoid cysts and facial milia.
Bazex-Dupré-Christol Syndrome	BCC	It is a rare condition characterized by follicular atrophoderma of the hands and feet, hypotrichosis, localized hypohidrosis, epidermoid cysts and multiple BCCs developed during the second decade of lifer showing a trichoepithelioma-like histology. The inheritance pattern is often X-linked.
Rombo Syndrome	BCC	Patients have an atrophoderma vermiculatum-like appearance on the cheeks with evidence of sweat duct proliferation. They often suffer of hypotrichosis, blepharitis, peripheral erythema, trichoepithelioma and skin cancer.

**Table 2:** Clinical and histologic risk factors associated with squamous cell carcinoma (NCCN Guidelines).

**Clinical Features**	**LOW RISK**	**HIGH RISK**
Site and Size	Area L; <20 mm Area M; <10 mm	Area L; ≥20 mm Area M; ≥10 mm Area H;
Margins	Well defined; R0	Undefined or R1
Immune Suppression	Absence	Presence
Exposition to radiotherapy or chronic inflammatory process	Absence	Presence
Rapid growth	Absence	Presence
Neurological symptoms	Absence	Presence
**Histology**	**LOW RISK**	**HIGH RISK**
Grading	G1 or G2	G3
Adenoid-squamous, Adenoid-cystic, Desmoplastic, Metaplastic, Carcinosarcoma	Absence	Any Variant
Thickness or level of invasion	≤6 mm and no subcutaneous invasion	>6 mm or subcutaneous invasion
Perineural, lymphatic or vascular invasion	Absence	Presence
